# Application of an OFA strategy to ERAS in a 102-year-old patient undergoing colon cancer surgery: A case report

**DOI:** 10.1097/MD.0000000000034431

**Published:** 2023-07-21

**Authors:** Jingwei Dai, Mengya Yang, Shanliang Li

**Affiliations:** a Department of Anesthesiology, Hainan Wanning People’s Hospital, Wanning Hainan, China.

**Keywords:** case report, enhanced recovery after surgery, opioid-free anesthesia, quadratus lumborum block, super-elderly patient

## Abstract

Colorectal cancer is the third most common cancer and the second leading cause of cancer-related deaths worldwide. Opioid-free anesthesia (OFA) is an opioid-sparing technique that focuses on multimodal or balanced analgesia, relying on non-opioid adjuncts and regional anesthesia. Enhanced recovery after surgery (ERAS) protocols, often under the auspices of a perioperative pain service, can help guide and promote opioid reduced and OFA, without negatively impacting perioperative pain management or recovery. Ultrasound-guided regional nerve block is currently a good option for OFA due to anesthesiologists’ mastery of ultrasound techniques. The safety of the OFA strategy for quadratus lumborum block (QLB) + transversus abdominis plane block (TAP) in the super-elderly patients has not been reported and remains unclear. We report a case of OFA anesthesia in a super-elderly patient with colon cancer.

**Patient concerns:**

A 102-year-old female was admitted to the hospital due to “abdominal pain for a week” and received conservative treatment for more than 20 days, with poor results.

**Diagnoses:**

The patient was diagnosed with colorectal cancer associated with bronchiectasis and infection, multiple nodules in the right lower lung, and sinus arrhythmia.

**Interventions:**

As the patient was a super-elderly patient with multiple diseases, we used an OFA strategy with general anesthesia combined with QLB and TAP.

**Outcomes:**

The patient awakened quickly and completely after surgery, and extubation was successful 2 min after surgery without anesthesia complications, which is in line with the concept of ERAS.

**Lessons:**

The OFA strategies of ultrasound guidance quadratus lumborum block (Ul-QLB) and ultrasound guidance transversus abdominis plane block (Ul-TAP) may be safe and effective for ERAS in super-elderly patients with colorectal cancer surgery.

## 1. Introduction

Enhanced recovery after surgery (ERAS) attenuates the stress response to surgery in the perioperative period and hastens recovery. ERAS decreased pain scores and opioid use without increasing the length of hospital stay or readmission.^[1]^ The broad and high-dose use of opioids has revealed its limitations: less efficacy on pain during movement, dose-dependent side effects, which can be very disabling for the patient and delay postoperative rehabilitation, dose-dependent hyperalgesia paradoxical source of acute and chronic pain, immunomodulation that may have a negative impact on infectious or cancerous pathologies and doubt about possible neurotoxicity. Opioid-free anesthesia (OFA) is a multimodal anesthesia strategy that combines multiple non-opioid drugs and/or techniques to achieve high-quality anesthesia without opioid.^[2]^ Ultrasound-guided regional nerve block is currently a good option for OFA, owing to the mastery of ultrasound techniques by anesthesiologists.

## 2. Case description

A 102-year-old female (155 cm, 59 kg) was admitted to the hospital due to “abdominal pain for a week” and received conservative treatment for more than 20 days, with poor results. Her medical history included chronic bronchitis, bronchiectasis with infection, hypoxemia, regular cough, and expectoration. She denied a history of surgery, anesthesia, or medication. ASA: III, ECG: Sinus arrhythmia, cardiac function grade: II, Goldman score:11. Her airway evaluation results were normal; the modified Mallampati classification was II, and the Arozullah score was 26. The laboratory test results were as follows; K^+^ 3.40 mmol/L GLU 7.76 mmol/L, TP 52.80 g/L, ALB 34.30 g/L, GLB18.50 g/L, TBA 27.84 μmol/L; Blood gas analysis: pH 7.44, PaO_2_ 79.50 mm Hg, PCO_2_ 33.60 mm Hg, FCOHb 3.30%, TCO_2_ 23.50 mmol/L, O_2_CT6.50 mmol/L, RI 0.47, Alveolar-arterial oxygen difference 37.20 mm Hg; Blood routine: Hb 101.00 g/L, Hct 32.90%; BNP 931.00 pg/L. 5.CRP 13.41mg/dL. Coagulation and renal function tests were normal. Chest CT revealed left inferior lobe bronchiectasis with infection, right inferior lobe honeycomb changes, aortic and coronary calcium plaques, and bilateral pleural thickening.

In the operating room, initial routine monitoring showed sinus arrhythmia with a heart rate of 85beats per minute, blood pressure of 147/55 mm Hg, respiratory rate of 25 per minute, and 97% pulse oxygen saturation (S_P_O_2_) on room air. The ECG, NBP, S_P_O_2_, T, RR, urine volume, bispectral index, and surgical pleth index (SPI) values were monitored. Dexmedetomidine 15 μg was pumped within 10 minutes, penehyclidine hydrochloride 0.6 mg was injected intramuscularly, and tropisetron (5 mg) was injected intravenously after opening the channel.

**Fig. 1.**
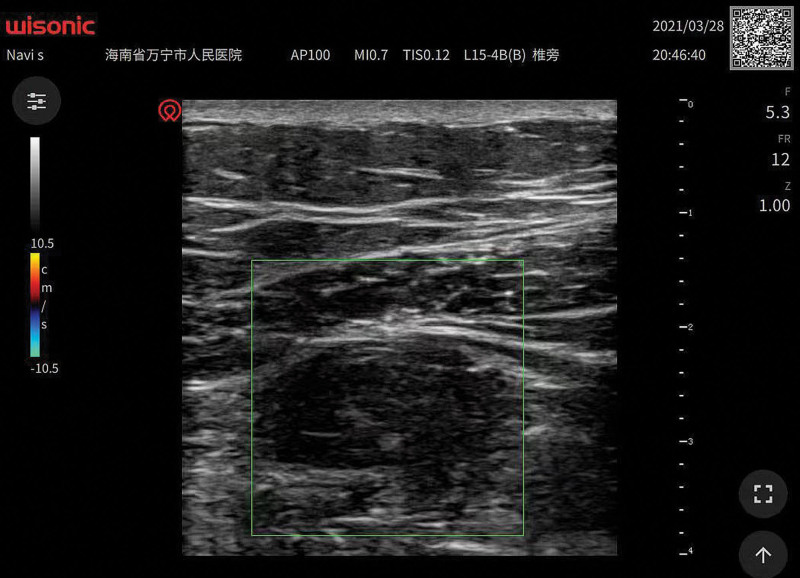
High-frequency probe QLB and TAP block path in supine position: SF = subcutaneous fat, LD = latissimus dorsi muscle, ES = erector muscle, SPI = serratus posterior inferior muscle, EO = external abdominal oblique muscle, IO = internal abdominal oblique muscle, TA = transversal abdominis muscle, TAP = transversus abdominis plane block, QL = quadratus lumborum, QLB = quadratus lumborum block, PM = psoas major muscle, AC = abdominal cavity, N1 = puncture path of quadratus lumborum, N2 = puncture path of transversal abdominal muscle, Green square = Doppler window.

After the patient was sedated, the 1-point method was used to perform ultrasound guidance quadratus lumborum block (Ul-QLB) via the anterior approach under local anesthesia in the supine position. The puncture needle was then withdrawn subcutaneously to perform the ultrasound guidance transversus abdominis plane block (Ul-TAP) (Fig. 1). Each block point was injected with 0.2% ropivacaine + dexamethasone 1.25 mg + 0.9% NS 20 mL, a total of 80 mL in 4 block points. After the block, the dermatomes of the sensory block at the 15th minutes were evaluated using pinprick.^[3]^

After induction with target-controlled infusion of propofol 3 μg/mL and intravenous cisatracurium (10 mg), an ID 7.0 endotracheal tube was inserted. A low tidal volume lung protection strategy was adopted: V_T_300 mL, PEEP6 cm H_2_O, RR 15 bpm, I/E 1:2.5, oxygen flow rate 2 L/minute, and P_ET_CO_2_ 35 to 45 mm Hg. Bispectral index values of 40 to 60 and SPI values of 30 to 50 were maintained. When the skin was sutured, propofol was stopped, intravenous flurbiprofen 50 mg was administered, and postoperative controlled intravenous analgesia was administered: esketamine 50 mg + flurbiprofen 200 mg + dexmedetomidine 50 ug + 0.9% NS to 100 mL, at a pump rate of 2 mL/hour. Anesthesia lasted for 260 minutes, and surgery lasted for 213 minutes with intermittent intravenous cisatracurium. Lactated Ringer’s solution (900 mL), succinylated gelatin (1000 mL), plasma (600 mL), suspended red blood cells 4u, blood loss (700 mL), urine volume (500 mL), and intraoperative vital signs.

At the end of surgery, the tidal volume of spontaneous breathing was approximately 300 mL, and the tracheal tube was removed for 2 minutes.

after full sputum suction. The S_P_O_2_ for oxygen inhalation through the mask was maintained at > 95%, and the patient was able to respond to calls, move limbs and breathe deeply. Half an hour later, the patient was transferred to the ICU under monitoring and an oxygen mask, and no complications of anesthesia were observed. Physical examination in ICU: T 36.3°C; P 88 bpm; RR 23 bpm; blood pressure 103/57mm Hg; S_P_O_2_ 97%; visual analogue scale 2 points; bilateral pupils, large and round; sensitivity to light reflex. Double lungs breathing thick, no dry or wet rales were heard. The heart rate was even and no pathological murmurs were heard in the auscultation area of each valve. Written informed consent was obtained from the patient’s son for the publication of this case report.

## 3. Discussion

ERAS protocols were first used in colorectal surgery.^[4]^ Increasing data suggest that the immunosuppressant effects of anesthesia can be circumvented by avoiding opioids and volatile anesthetics.^[5]^

Regional anesthesia/analgesia is, of course, the best technique to reduce or avoid intraoperative opioids. Indeed, the blockage of nociceptive afferences is perfectly ensured by regional anesthesia/analgesia, and its benefits have long been proven in the literature.^[6]^ The quadratus lumborum block (QLB) may be used to provide postoperative analgesia in the abdominal and pelvic regions.^[7]^

Transversus abdominis plane block (TAP) cannot provide visceral analgesia. Human and animal studies have shown that QLB provides somatic and visceral analgesia.^[8,9]^ Compared with TAP, QLB covers a wider range of terrain (Th7–Th12 vs Th10–Th12) and produces a longer painless state than TAP (24–48 hours vs 8 to 12 hours). The QLB produces a broad distribution of local anesthetics, resulting in a large area of sensory inhibition of (T7 through L1 in most cases). Therefore, the QLB and TAP can complement each other in terms of the block plane, block time, and nerve block type. Attention: Ipsilateral Ul-QLB was performed first, followed by Ul-TAP; otherwise, the liquid injected into the transversus abdominis plane would have affected the ultrasound image and visual field of the quadratus lumborum.

Studies have shown that SPI can reflect the regulatory activity of sympathetic nerves on the cardiovascular system and evaluate the response of the central nervous system to pain “nociception” during general anesthesia.^[10]^ Studies have shown that it is safe to inject 20 mL 0.375% ropivacaine (total dose 225 mg) at each injection site for parasacral block combined with anterior quadratus lumborum and psoas compartment blocks.^[11]^ The OFA strategy of Ul-QLB had little effect on inflammatory factors in patients undergoing lower abdominal surgery, and extubation and awake times were shorter than those in the control group.^[12]^

In this case, consciousness and breathing recovered quickly, vital signs were stable during the operation, and extubation was successful 2 minutes after the operation. The OFA strategy of Ul-QLB and Ul-TAP may be safe and effective for ERAS in super-elderly patients undergoing surgery for colon cancer.

## Author contributions

**Data curation:** Yang Mengya.

**Visualization:** LI Shanling.

**Writing – original draft:** DAI Jingwei.

**Writing – review & editing:** DAI Jingwei.

## References

[1] Brown ML Simpson V Clark AB. ERAS implementation in an urban patient population undergoing gynecologic surgery. Best Pract Res Clin Obstet Gynaecol. 2022;85(Pt B):1–11. 10.1016/j.bpobgyn.2022.07.009 36031533

[2] Beloeil H. Opioid-free anesthesia. Best Pract Res Clin Anaesthesiol. 2019;33:353–60. 31785720 10.1016/j.bpa.2019.09.002

[3] Zhu M Qi Y He H. Effect of quadratus lumborum block on postoperative cognitive function in elderly patients undergoing laparoscopic radical gastrectomy: a randomized controlled trial. BMC Geriatr. 2021;21:238. 33836651 10.1186/s12877-021-02179-w PMC8033654

[4] Ljungqvist O Scott M Fearon KC. Enhanced recovery after surgery: a review. JAMA Surg. 2017;152:292–8. 28097305 10.1001/jamasurg.2016.4952

[5] Hurtado C Bendure J Bennetts P. Anesthetic and analgesic influence on cancer recurrence and metastasis. AANA J. 2021;89:221–6. 34042573

[6] Lavand’homme P De Kock M Waterloos H. Intraoperative epidural analgesia combined with ketamine provides effective preventive analgesia in patients undergoing major digestive surgery. Anesthesiology. 2005;103:813–20. 16192774 10.1097/00000542-200510000-00020

[7] Dhanjal S Tonder S. Quadratus Lumborum Block. In: StatPearls. Treasure Island (FL): StatPearls Publishing; 2022. 30725897

[8] Viscasillas J Sanchis-Mora S Burillo P. Evaluation of quadratus lumborum block as prt of an opioid-free anaesthesia for canine ovariohysterectomy. Animals (Basel). 2021;11:3424. 34944201 10.3390/ani11123424 PMC8697988

[9] Wang H Deng W Zhu X. Perioperative analgesia with ultrasound-guided quadratus lumborum block for transurethral resection of prostate. Medicine (Baltim). 2021;100:e28384. 10.1097/MD.0000000000028384 PMC8702260 34941168

[10] Ince B Zuhour M Yusifov M. The impact of surgical procedures during septorhinoplasty on the intraoperative pain response. Aesthet Surg J. 2021;41:NP1421–6. 34031694 10.1093/asj/sjab234

[11] Seidel R Barbakow E Schulz-Drost S. Surgical treatment of proximal femoral fractures in high-risk geriatric patients under peripheral regional anesthesia: a prospective feasibility study [in English]. Anaesthesist. 2021;70:1022–30. 33713157 10.1007/s00101-021-00935-6

[12] Dai J Lin S Xu Z. Effect of ultrasound-guided quadratus lumborum block on deopiation in supine position. Chongqing Med. 2023;52:686–91.

